# Constant learning - an interview with Lilian Kabeche on setting up an inclusive and welcoming lab

**DOI:** 10.1038/s42003-022-03599-z

**Published:** 2022-06-29

## Abstract

Dr. Lilian C. Kabeche is an Assistant Professor of Molecular Biophysics and Biochemistry at Yale University, USA. She received her PhD from Dartmouth College working on kinetochore-microtubule attachments in Duane Compton’s laboratory, followed by a postdoctoral training on the DNA damage response in Lee Zou’s group at Harvard Medical School. Lilian started her independent career in 2019.

**Figure:**
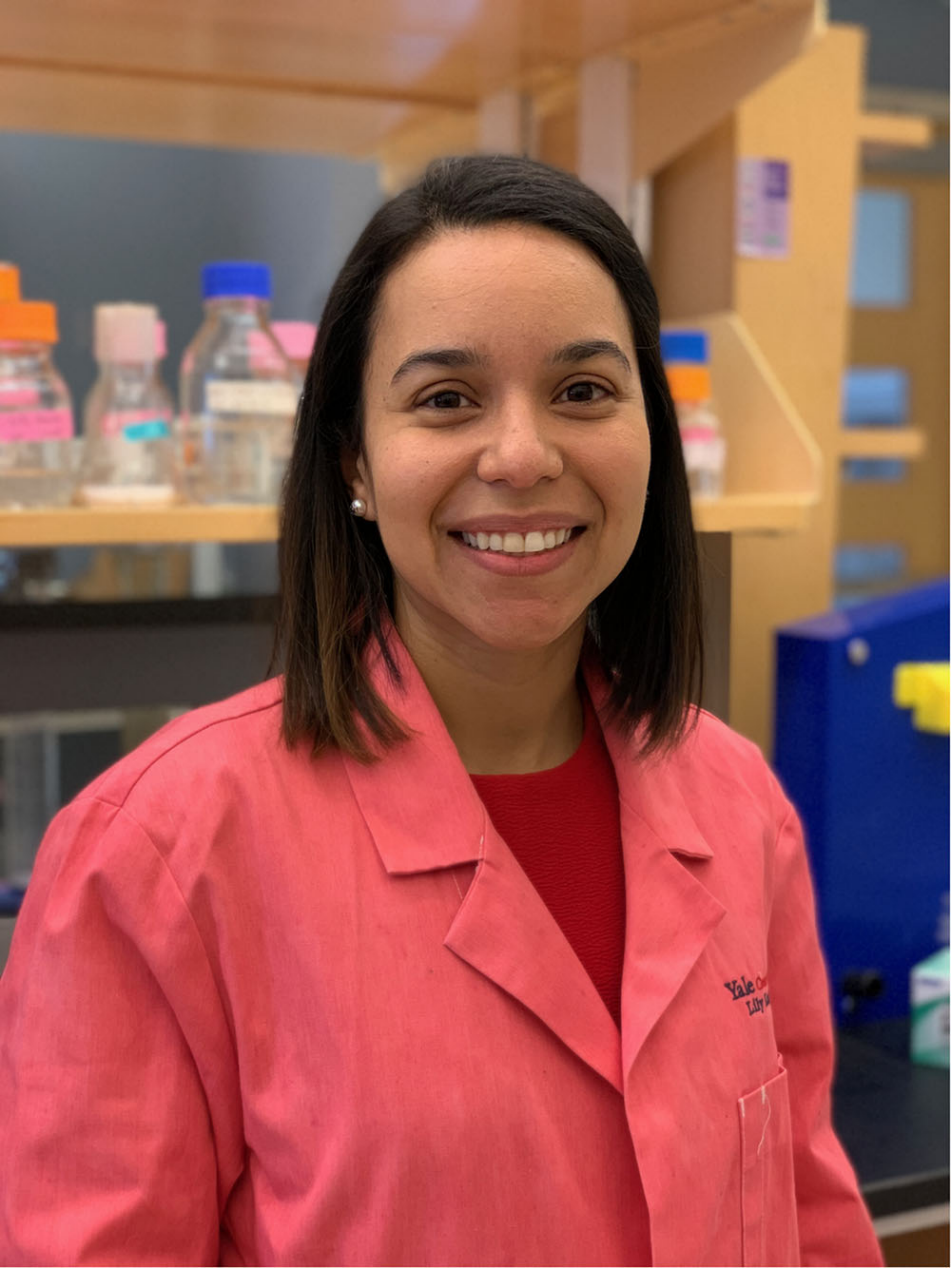
Lilian Kabeche

Do you have an inspiration or ‘heureka’ moment that inspired you to become a scientist?

I have always been fascinated by science but I didn’t really know what I wanted to do after college until I started research as an undergraduate in Dr. Arba Ager’s laboratory (University of Miami, USA). Having immigrated from Venezuela, I didn’t know doing research was a career – it’s not a common profession there, especially for women. In the Ager lab I encountered Merida, a research associate and the first Hispanic female scientist that I had ever met and it allowed me to see myself as a scientist and she empowered me to apply for graduate school.

Your research career has been centered around cell cycle checkpoints. What fascinates you about the Spindle Assembly Checkpoint and DNA damage checkpoint?

I don’t know if it’s the checkpoints themselves or the idea that there is still so much to learn about the proteins that make up the cell cycle checkpoints and pathways. I am perpetually amazed at the many functions of proteins that we often ignore, overlook or avoid because we label proteins as being part of a specific checkpoint or pathway. I am lucky, because for all of my work, I think I have been the person that was at the right place and the right time and had the naivety to ask a ‘crazy’ research question [about ATR’s role in mitosis] at the time and thankfully I wasn’t completely wrong.

ATR turns out to be a regulator at/ for several stages of the cell cycle and mitosis. What’s next for this “swiss army knife” kinase?

ATR is amazing! My lab has uncovered several new functions both in interphase and in mitosis that we are excited to share as soon as we have enough data! What I think is also fascinating is that ATR isn’t an outlier, it is just an example of this really interesting phenomenon of proteins performing multiple functions depending on the context - whether that’s their localization, the cell cycle or something else.

Can you tell us about the challenges you had to face as a PI of a young lab due to the shutdown caused by the pandemic?

I faced a lot of challenges of any working parent during the pandemic. I had to figure out how to balance a newborn and work and there was a lot of fear and a lot of questions like “Can I keep my lab from falling apart”? However, I am in a very privileged population that was able to work from home during the lockdown and our job offers flexibility to shift schedules around to be able to take care of a baby and do work. I know many other people didn’t have either of these luxuries.

How did you support the well-being of your lab members, and your own?

We have a wonderful group in my lab and I supported their well-being as much as they supported mine. In addition to journal clubs and one-on-one meetings, we also met every weekday for lunch via zoom and had our “sanity checks” where we would just talk: about our feelings, anxieties, the best/worst part of our day, and sometimes plotted as to how to break into the lab to do science (for the record, we never ended up going through with these plans!). These check-ins gave us a sense of normality. Also, I was really transparent about my struggles during this time, which I think empowered my lab members to say when they were struggling too so that they didn’t have to do this alone.

What characteristics do you look for when you’re recruiting new group members?

Someone who is enthusiastic, driven, and seriously loves science. I think that the techniques we do in the lab can be taught. If you are enthusiastic and have the drive to power through the hard parts of research, then the world is yours and you can learn and achieve anything. Love of science is what keeps someone going and motivated when it gets tough. I am really proud of the lab environment – the team members work well together and support each other - so it’s important that whoever joins the lab is able to maintain that supportive and enriching lab environment.

How important is diversity to you and what are the impediments for creating inclusive, equitable research labs and practices?

This is something that my lab and I take very seriously. We have an open dialog about this and really try to put into action how much we value inclusivity and equitability. We are all very different in the lab, with different skill sets, backgrounds and experiences and we embrace and celebrate each other’s differences. We try our best to improve our understanding and to create an inclusive and equitable research environment. Constant learning is key to maintain this culture. It takes practice and work and requires continual learning and listening (and sometimes listening to things that make you uncomfortable) to each other. It takes time and sometimes money to get that done and an evolving process as policies we enact and changes we make may create other problems that need to be addressed. It’s not enough to have one set of sweeping changes and then call it a day.

What can the research community and academic publishers do to champion diversity and inclusivity?

We need to continue increasing and retaining diverse representation at all levels of academia and the research community. To do so, we need to recognize where we’re failing to implement this—in our own lab, institution, or research field, and actively address it.

Could you tell us an interesting fact about yourself that people wouldn’t know by looking at your CV?

I am an avid gardener and while I am allergic to cats, I have two of them (very fluffy)! I also take full credit and blame for convincing my two sisters to pursue PhDs in the biological sciences as well—one of which completed hers in the lab next door to my graduate lab!

